# Women’s recall of health care provider counselling on gestational weight gain (GWG): a prospective, population-based study

**DOI:** 10.1186/s12884-019-2283-x

**Published:** 2019-04-25

**Authors:** Angela Vinturache, Anika Winn, Cynthia Mannion, Suzanne Tough

**Affiliations:** 1 Department of Paediatrics, Cumming School of Medicine, University of Calgary, Calgary, Alberta Canada; 2 Faculty of Health Sciences, Cumming School of Medicine, University of Calgary, Calgary, Alberta Canada; 3 Faculty of Nursing, Cumming School of Medicine, University of Calgary, Calgary, Alberta Canada; 4 Department of Community of Health Sciences, Cumming School of Medicine, University of Calgary, Calgary, Alberta Canada; 5 Department of Obstetrics & Gynaecology, St. George’s University Hospitals NHS Foundation Trust, London, UK

**Keywords:** Gestational weight gain, Obesity in pregnancy, Prenatal counselling, Women experiences, Health care provider, Prenatal counselling, Nutrition, Lifestyle

## Abstract

**Background:**

Prenatal care has been validated to provide medical and educational counselling intended to reduce the risk of adverse pregnancy conditions and improve the maternal and fetal outcomes. Prenatal targeted information regarding nutrition, lifestyle, and weight gain is predictive of meeting Institute of Medicine (IOM) 2009 gestational weight gain (GWG) guidelines. There is limited information about women’s experiences with these prenatal counselling domains, particularly in women who do not meet GWG recommendations. The objective of this study was to evaluate the impact of women’s recall of prenatal counselling and its effect on meeting their GWG within guidelines in a prospective, community-based pregnancy cohort.

**Methods:**

A sample of 2909 women with singleton pregnancies was drawn from the prospective community-based pregnancy cohort All Our Families from Alberta, Canada. Women were stratified into three GWG groups, adequate, inadequate, and excessive GWG, based on pre-pregnancy BMI and the adherence to the Institute of Medicine weight gain in pregnancy guidelines. At less than 25 and 34 to 36 weeks’ gestation, maternal socio-demographic information and women’s recall of prenatal counselling experiences was collected through self-administered questionnaires. Multivariate logistic regression analyses tested GWG strata impact on women’s recall of the prenatal counselling advice in eight domains of nutrition, lifestyle, and weight management during pregnancy.

**Results:**

Adequate GWG was reached by 35.9% of women, 46.5% gained excessive and 17.6% gained inadequate weight. Women who were overweight and obese prior to pregnancy were more likely to gain excessive weight than women who were normal weight (OR 3.3, 95% CI 2.6–4.1; and OR 2.9, 95% CI 2.1–3.9, respectively). Most women reported having no difficulties in finding prenatal care, felt comfortable with their health care provider and were satisfied with the answers received. There was no difference in the recall of prenatal advice received in any of the eight domains of prenatal counselling assessed among women with appropriate and non-optimal GWG.

**Conclusion:**

Women with adequate and non-optimal GWG received comparable prenatal counselling on nutrition, weight gain, and lifestyle modifications. There remain missed opportunities in targeting prenatal counselling advice to women at risk for suboptimal or excessive GWG.

**Electronic supplementary material:**

The online version of this article (10.1186/s12884-019-2283-x) contains supplementary material, which is available to authorized users.

## Background

Non-optimal gestational weight gain (GWG), whether excessive or inadequate, is associated with increased risk of poor maternal and fetal outcomes [1–7]. Maternal obesity and excessive GWG are associated with pregnancy complications (preeclampsia, gestational diabetes, fetal macrosomia), increased risk of interventions at delivery (induction of labour, shoulder dystocia, caesarean section, operative delivery), and increased perinatal morbidity and mortality [1–4]. Inadequate GWG increases the risk of preterm birth, low birth weight, and poor fetal development [5, 6]. GWG is a complex biological phenomenon, and the mechanisms through which non-optimal GWG mediates pregnancy and fetal adverse effects, including genes, environment, independent intergenerational programming effects, and a life-long persistent metabolic phenotype are not well understood [7–11]. Thus, pre-conception healthy weight and achieving appropriate GWG in pregnancy are important in promoting positive short- and long-term maternal and fetal outcomes [12].

As GWG is a modifiable risk factor, pregnancy represents an window of opportunity to initiate preventative measures [13]. Pregnant women may be more receptive and motivated for behavioral and lifestyle changes during this period given concern for their baby’s health [14]. Prenatal care has been endorsed to identify mothers at risk and provide them with targeted information regarding nutrition, lifestyle, and weight gain during pregnancy. Guidelines and recommendations have been developed to aid health professionals in counselling women regarding the potential risks and adverse outcomes of obesity and non-optimal GWG. The guidelines endorse individualized measures to improve lifestyle behaviours, dietary intake, and physical activity prior to and during pregnancy to promote healthy weight, gain weight within the guidelines, and return to a healthy weight after delivery [15–17]. There is limited evidence, however, regarding women’s knowledge of the risks of obesity, weight gain during pregnancy, and the consequences of excess body weight on their own health and that of the offspring, their awareness of GWG guidelines, or effective management strategies.

Studies indicate that the quality and quantity of information pregnant women receive from health professionals varies widely [18]. Provision of information on obesity guidelines and appropriate weight gain by health care professionals to pregnant women is predictive of women meeting GWG guidelines [19]. Nevertheless, one-time counselling and advice for healthy eating and exercise may be insufficient to help most women, particularly those with multiple risk factors or other health concerns. Ongoing conversation over the course of pregnancy and adjusted, targeted interventions may be more effective [20, 21]. There is limited information, however, about women’s experiences with effective prenatal counselling, particularly in women who do not meet GWG recommendations. Very little is known about what information is significant and memorable and how women receive and retain this information that influences GWG goals and healthy pregnancy lifestyle.

In this study we aimed to evaluate the impact of women’s recall of prenatal counselling on meeting GWG guidelines in a prospective cohort of low risk pregnancies.

## Methods

### Study design

Data for this study were drawn from the All Our Families (AOF), a prospective, community-based, low risk pregnancy cohort from Calgary, Alberta, Canada.

The study methodology including setting, population, eligibility, and recruitment has been described in detail elsewhere [22]. Briefly, the AOF is a prospective cohort study designed to examine the determinants of maternal, infant, and child outcomes and identify barriers and facilitators in health care utilization. The AOF study enrolled pregnant women who received prenatal care in primary care offices from the Calgary metropolitan area, between May 2008 and December 2010. Women were eligible if older than 18 years, able to read and write in English, and less than 24 weeks pregnant at the time of recruitment. Structured questionnaires were administered at less than 25 weeks, and at 32 to 36 weeks gestation. The data collected included information on women’s demographics (mother’s age, marital status, education, race, and socioeconomic status), pregnancy and health history, lifestyle (cigarette smoking, substance use prior and during pregnancy, physical activity), and health care utilization, including women’s experiences with prenatal counselling.

The information from questionnaires was linked via unique identifier (health care number) to the electronic health records for labour and delivery. Health records contained additional details on pregnancy and delivery not captured by the surveys (onset of labour, delivery method, child’s sex, birth weight, gestational age). The dataset also included information on number of prenatal visits and type of health care provider. For this study, women with multiple pregnancies, non-cephalic presentations, preterm deliveries, or missing information regarding prenatal counselling were excluded from the analysis. This resulted in the inclusion of 2909 women with low-risk pregnancies who delivered at term, singleton, live-born infants.

The questionnaires explored the level of counselling women received in eight different domains of prenatal care following the Society of Obstetricians and Gynecologists of Canada (SOGC) Clinical Practice Guidelines [23]. Women were asked whether they had been counselled on nutrition, vitamin and mineral supplements, alcohol consumption, exercise and active living, appropriate weight gain throughout pregnancy, working during pregnancy, non−/prescription drug use, and cigarette smoking, including exposure to second hand smoke. The survey also included questions exploring the level of comfort and satisfaction of the patients during the encounter with their health care provider: i) Do you feel comfortable asking a question to your prenatal care provider about your pregnancy? ii) Are you able to get an answer that you are satisfied with and can act on if necessary? The questions were dichotomous, with “yes or no”-type answers.

### Variables and statistical analyses

Maternal body mass index (BMI) was calculated from self-reported pre-pregnancy weight and height. Pre-pregnancy BMI was categorized according to the World Health Organization’s definition into four groups: underweight (BMI < 18.50 kg/m^2^), normal weight (18.50–24.99 kg/m^2^), overweight (25.00–29.99 kg/m^2^), and obese (BMI ≥ 30.00 kg/m^2^) [24]. Pregnancy weight gain was determined using the mother’s weight prior to delivery (at the time of the last administered survey) subtracted from the pre-pregnancy weight. Pre-pregnancy BMI allowed subsequent stratification of women into three GWG groups based on the adherence to the Institute of Medicine (IOM) 2009 guidelines recommendations on weight gain in pregnancy [12]: appropriate, inadequate and excessive. The number of prenatal visits was examined and categorized as ‘less than 6’ (≤ 6) and ‘7 or more’ (≥ 7). Frequency of exercise per week was classified as ‘two times or less’ or ‘three times or more’. Cigarette smoking and other substance use during pregnancy, were coded as ‘yes’ or ‘no’. Gestational age was determined by ultrasound in the first trimester of pregnancy [25].

Descriptive statistics, proportions, means and standard deviations, were generated for continuous and categorical variables for the sample characteristics and outcomes. Bivariate analyses (chi-square, regression) assessed the relationship between women’s socio-demographic and pregnancy characteristics and maternal GWG category.

Multivariable logistic regression analysis evaluated the association between GWG category, and the type of prenatal counselling received, controlling for confounding variables: age, ethnicity, education, income, parity, time in Canada. Odds ratios and 95% confidence intervals were calculated for all models, which included only significant predictor variables for the considered outcome. All statistical analyses were performed using the IBM SPSS for Windows statistical software package, version 22.0 (IBM SPSS, Chicago, IL).

## Results

From 2909 women in the study, 1808 (62.2%) were normal weight, 647 (22.2%) were overweight, and 322 (11.1%) were obese prior to pregnancy. Approximately one third of women, 35.9%, gained the appropriate amount of weight, almost half, 46.5%, gained more, and 17.6% gained less weight when compared to IOM 2009 recommendations for each BMI category. Overweight and obese women were more likely to gain excessive weight during pregnancy than normal weight women (unadjusted odds ratio, uOR 3.3, 95% CI 2.6–4.1 for overweight and uOR 2.9, 95% CI 2.1–3.9, respectively; *p* < 0.001). (Additional file 1: Figure S1).

Socio-demographic characteristics, lifestyle, and obstetrical details of participants were stratified by GWG (Table 1). The average age of the participants was 30.7 years (SD 4.6 years) and the mean BMI was 24.2 (SD 5.0 kg/m^2^). The majority of women were Caucasian (79.5%), had attained higher education (post-secondary and higher) (90.2%) and had a household income higher than 60 K CAD (the province of Alberta annual median income of a urban family with children at the time of the study) (83.4%). Women who gained excessive weight were more likely to have been born or have lived in Canada > 5 years (92.2%) and be employed in full-time jobs (72.8%).

**Table 1:** Characteristics of participants included in the study, stratified by gestational weight gain (GWG)^a^

Characteristics	All women		Adequate GWG	Inadequate GWG	Excessive GWG	*p*-value
	Total sample (N)^b^	n (%)	n (%)	n (%)	n (%)	
	2909		1045 (35.9)	511 (17.6)	1353 (46.5)	
Socio-demographic and anthropometric characteristics						
Maternal age (years)	2843					
Mean ± SD		30.7 ± 4.5	30.8 ± 4.6	30.7 ± 4.6	30.6 ± 4.4	0.639
≤ 35-year-old		2277 (80.0)	816 (80.3)	394 (79.0)	1067 (80.3)	0.783
> 35-year-old		566 (20.0)	200 (19.7)	105 (21.0)	261 (19.7)	
Ethnicity	2902					0.070
White/Caucasian		2307 (79.5)	843 (80.7)	386 (75.8)	1078 (79.9)	
Other		595 (20.5)	201 (19.3)	123 (24.2)	271 (20.1)	
Time in Canada	2893					0.006*
Born/ lived in Canada ≥ 5years		2626 (90.8)	943 (90.6)	442 (87.4)	1241 (92.2)	
Lived in Canada <5 years		267 (9.2)	98 (9.4)	64 (12.6)	105 (7.8)	
Education	2904					0.947
High-school or less		285 (9.8)	100 (9.6)	50 (9.8)	135 (10.0)	
Some or completed post-secondary		2619 (90.2)	943 (90.4)	460 (90.2)	1216 (90.0)	
Household Income	2816					0.172
< $60 000		467 (16.6)	153 (15.2)	94 (19.0)	220 (16.8)	
≥ $60 000		2349 (83.4)	856 (84.8)	402 (81.0)	1091 (83.2)	
Employment status	1933					0.042*
Employed full time		1340 (69.3)	496 (66.5)	215 (65.7)	656 (72.8)	
Employed part-time		497 (25.7)	198 (28.1)	95 (29.1)	204 (22.6)	
Casual employment		96 (5.0)	38 (5.4)	17 (5.2)	41 (4.6)	
Pre-pregnancy weight (kg) (mean ± SD)	2909	70.0 ± 14.2	65.9 ± 12.5	68.1 ± 16.0	73.8 ± 13.7	<0.001*
Pre-pregnancy BMI (kg/m^2^) (mean ±SD)	2909	24.2 ± 5.0	24.1 ± 4.3	24.9 ± 5.6	26.7 ± 4.6	<0.001*
^a^Maternal pre-pregnancy BMI (kg/m^2^)	2909					<0.001*
Underweight (<18.5 kg/m^2^)		132 (4.5)	68 (6.5)	29 (5.7)	35 (2.6)	
Normal weight (18.5- 24.9 kg/m^2^)		1808 (62.2)	761 (72.8)	356 (69.7)	691 (51.1)	
Overweight (25-29.9 kg/m^2^)		647 (22.2)	147 (14.1)	56 (11.0)	444 (32.8)	
Obese (≥30.0 kg/m^2^)		322 (11.1)	69 (6.6)	70 (13.7)	183 (13.5)	
Obstetrical characteristics						
Parity n (%)	2893					0.220
Nulliparous		1400 (48.4)	497 (47.6)	234 (45.8)	669 (50.0)	
Multiparous		1493 (51.6)	547 (52.4)	277 (54.2)	669 (50.0)	
Gestational age at delivery (mean ±SD)	2737	39.2 ± 1.6	39.3 ± 1.7	39.0 ± 1.7	39.2 ± 1.6	0.053
Preterm birth rate n (%)	2909					0.357
< 37 weeks GA		148 (5.4)	48 (4.8)	31 (6.6)	69 (5.5)	
≥ 37 weeks GA		2589 (94.6)	955 (95.2)	439 (93.4)	1195 (94.5)	
Mode of delivery n (%)						
Vaginal (spontaneous or assisted)	2754	2089 (75.9)	363 (17.4)	787 (37.7)	939 (44.9)	0.045*
Caesarean Section		665 (24.1)	109 (16.4)	221 (33.2)	335 (50.4)	
Elective C-section		323 (12.8)	112 (12.3)	54 (12.4)	157 (13.3)	
Emergency C-section		331 (13.1)	111 (12.2)	51 (11.7)	169 (14.3)	
Type of labour	2424					
Spontaneous labour		1472 (60.7)	561 (64.4)	251 (60.5)	660 (57.9)	0.047*
Induction of labour		666 (27.5)	211 (24.3)	115 (27.7)	340 (29.8)	
No labour		286 (11.8)	97 (11.2)	49 (11.8)	140 (12.3)	
Lifestyle characteristics						
Exercise and active living in pregnancy	2908					0.001*
Frequency of exercise/week						
0 to 2 times		1620 (55.7)	554 (53.0)	262 (51.3)	804 (59.5)	
3 or more times		1288 (44.3)	491 (47.0)	294 (48.7)	548 (40.5)	
Comparison to frequency of exercise/week prior to pregnancy	2909					<0.001*
Less often		1774 (61.0)	634 (60.7)	264 (51.7)	876 (64.7)	
More often		178 (6.1)	59 (5.6)	30 (5.9)	89 (6.6)	
About the same		957 (32.9)	352 (33.7)	217 (42.5)	388 (28.7)	
Substance abuse in pregnancy						
Smoking	2701	302 (11.2)	101 (10.3)	38 (8.0)	163 (13.1)	0.006*
Alcohol consumption	2704	1331 (49.2)	500 (51.1)	213 (44.7)	618 (49.5)	0.075
Recreational drugs use	2905	112 (3.9)	33 (3.2)	11 (2.2)	68 (5.0)	0.006*

^a^BMI and GWG classified according to Institute of Medicine 2009 Guidelines and Health Canada Guideline

^b^May not add to *N* = 2909 participants due to missing data

*SD* standard deviation

*p-value < 0.05 significant

There were significant lifestyle differences between the three groups of women. Women who gained excessive weight were more likely to lead a less active lifestyle during pregnancy, exercising less often than prior to pregnancy as compared to women who gained the recommended GWG (*p* < 0.001). Furthermore, these women were more likely to smoke and use recreational drugs during pregnancy than women with adequate GWG (*p* = 0.006). There were no differences in parity, rates of preterm birth, or gestational age at delivery between women with adequate and non-optimal GWG. Women with excessive GWG were more likely to have induction of labour and be delivered by caesarean section (*p* = 0.047, and *p* = 0.045, respectively) than women with adequate GWG.

Prenatal care in women with adequate and non-optimal GWG is described in Table 2. More than 90% of women recalled having no difficulties in finding prenatal care services. Among the reasons cited by the women who reported difficulties in finding a care provider (10%), the most frequent motive was not finding a doctor or midwife who were currently accepting prenatal patients. Other reasons women reported were: lack of transportation to and from the clinic, lack of child care, stress, cultural beliefs, lack of awareness of such services being available, fear about pregnancy or delay in suspecting pregnancy. There were no differences between the GWG groups regarding the difficulties in obtaining prenatal care.

**Table 2:** Prenatal care in women with adequate, inadequate and excessive gestational weight gain (GWG)^a^

	Gestational Weight Gain				
	Total Sample (N)	Adequate GWG n (%)	Inadequate GWG n (%)	Excessive GWG n (%)	*p*-value
Difficulty obtaining prenatal care n (%)	2909				
Yes		56 (5.4)	34 (6.7)	64 (4.7)	0.253
No		989 (94.6)	477 (93.3)	1289 (95.3)	
Number of prenatal care visits (mean ± SD)	2849	8.2 ± 2.0	8.2 ± 2.2	8.3 ± 2.2	0.408
≤ 6 prenatal visits	672	234 (34.8)	128 (19.0)	310 (46.1)	0.471
> 7 prenatal visits	2177	789 (36.2)	371 (17.0)	1017 (46.7)	
Type of prenatal care provider					
Obstetrician	1116 (38.4)	360 (32.3)	209 (18.7)	547 (49.0)	0.005*
Family physician	1783 (61.3)	631 (35.3)	341 (19.1)	811 (45.6)	0.020*
Midwife	272 (9.4)	123 (45.3)	36 (13.2)	113 (41.5)	0.002*
Low Risk Maternity Clinic Doctor	1359 (46.7)	495 (36.4)	233 (17.1)	631 (46.5)	0.803
Walk-in Clinic Doctor	268 (9.2)	87 (32.4)	49 (18.2)	132 (49.3)	0.461
Other health care provider^b^	238 (8.2)	82 (34.5)	38 (15.9)	118 (49.6)	0.589
Proportion of women who felt comfortable asking for advice^c^	2823	1016 (99.2)	495 (99.0)	1312 (98.7)	0.498
Proportion of women who received satisfactory answer to their questions^c^	2671	971 (97.8)	470 (97.3)	1230 (96.9)	0.400
Domains of prenatal advice women recalled receiving advice^c^					
Nutrition	2018	721 (69.0)	357 (69.9)	940 (69.5)	0.935
Vitamins/mineral supplements	2495	920 (88.0)	435 (85.1)	1140 (84.3)	*0.029
Alcohol consumption	1417	522 (50.0)	234 (45.8)	661 (48.9)	0.302
Exercise and active living	1834	662 (63.3)	17.2 (61.6)	857 (63.3)	0.770
Appropriate weight gain	1930	681 (65.2)	337 (65.9)	912 (67.4)	0.505
Working during pregnancy	1430	516 (49.4)	234 (45.8)	680 (50.3)	0.224
Non−/prescription drugs	1777	647 (61.9)	318 (62.2)	812 (60.0)	0.539
Smoking (including second hand smoking)	1206	437 (41.8)	203 (39.7)	566 (41.8)	0.682

^a^ Gestational weight gain (GWG) was classified based on Institute of Medicine 2009 recommendations

^b^ Other health care providers refer to one or more of the following: high risk obstetrics or maternal-fetal medicine specialist, endocrinologist, dietitian, chiropractor, massage therapist, emergency medicine specialist, sonographer, alternative medicine specialist etc.

^c^ N represents the number of women who answered ‘yes’ to receiving advice in the specific domain of prenatal counselling

*p-value < 0.05 significant

The majority of women (97%) reported feeling comfortable in asking questions, receiving advice, and were satisfied (92%) with the answers from the health care provider. There were no differences in the level of comfort for asking questions or in the satisfaction with the answers received between the three groups of women (Table 2).

The average number of prenatal visits was 8 (range 1 to 12). Table 2 illustrates the average number of prenatal visits in underweight, normal weight, overweight, and obese stratified by GWG. There were no differences in the number of prenatal visits attended by the women from the three GWG groups. Women had prenatal visits with their family physician (61.3%), low-risk maternity clinic doctor (46.7%), and obstetrician (38.4%). Less than 10% of women had an appointment with a midwife. Women with non-optimal GWG were more likely to receive prenatal care from an obstetrician (uOR 1.3, 95% CI 1.09–1.5 for excessive GWG and uOR 1.3, 95% CI 1.05–1.6 for inadequate GWG, respectively) or a family physician (uOR 1.3, 95% CI 1.05–1.6 for women with inadequate GWG) and less likely to attend prenatal care with a midwife (uOR 0.6; 95% CI 0.6–0.8, and uOR 0.5; 95% CI 0.3–0.8, respectively) (calculated unadjusted odds ratio, uOR and 95% confidence intervals not shown in table). Approximately 8.2% (238) of women have also seen another type of health care provider at some point during their pregnancy, such as: nutritionist, endocrinologist, maternal-fetal medicine specialist, high-risk obstetrics specialist, chiropractor or massage therapist, homeopath, naturopath, emergency services doctor. The rate of accessing alternative health care providers was similar for women with adequate, inadequate, and excessive GWG.

Regarding the domains of prenatal counselling, most women recalled receiving advice on vitamins and mineral supplementation, and approximately two thirds on nutrition, exercise and active living, and appropriate weight gain. Less than half of women recalled advice on smoking and drinking in pregnancy. The Additional file 2: Table S1 shows women’s recall of prenatal counselling stratified by maternal demographic and obstetrical characteristics, including maternal pre-pregnancy BMI, age, education, ethnicity, income, parity, and number of prenatal visits, the covariates included in the multivariable models.

Table 3 shows unadjusted and adjusted association between GWG and domains of prenatal counselling. In unadjusted analyses, using adequate GWG as the reference category, there was similar recall of prenatal counselling in all domains among women with inadequate and excessive GWG. After controlling for covariates, multivariate analyses revealed no difference in the advice received on any of the prenatal counselling domains between women with inadequate and excessive GWG compared to women with adequate GWG.

**Table 3:** Odds Ratios for the relationship between women’s recall of prenatal advice and gestational weight gain

	Inadequate GWG				Excessive GWG			
	uOR	95% CI	aOR	95% CI	uOR	95% CI	aOR	95% CI
Felt comfortable asking for advice	0.78	0.3-2.4	0.74	0.2-2.6	1.28	0.5-3.5	1.49	0.5-4.5
Received satisfactory answer	0.82	0.4-1.6	0.83	0.4-1.7	1.18	0.6-2.2	1.20	0.6-2.3
Domains of prenatal counselling								
Nutrition	0.96	0.8-1.2	0.91	0.7-1.2	0.98	0.8-1.2	0.93	0.7-1.2
Vitamins/mineral supplements	1.29	0.9-1.7	1.31	0.9-1.8	0.94	0.7-1.2	0.88	0.6-1.2
Alcohol consumption	1.18	1.0-1.5	1.13	0.9-1.4	1.13	0.9-1.4	1.07	0.9-1.3
Exercise/active living	1.08	0.9-1.3	1.07	0.8-1.4	1.08	0.9-1.3	1.02	0.8-1.3
Appropriate weight gain	0.97	0.7-1.2	0.94	0.7-1.2	1.07	0.9-1.3	1.00	0.8-1.2
Working during pregnancy	1.16	0.9-1.4	1.11	0.9-1.4	1.20	1.0-1.5	1.13	0.9-1.4
Non-/prescription drugs	0.99	0.8-1.2	0.94	0.7-1.2	0.91	0.7-1.1	0.84	0.7-1.0
Smoking during pregnancy	1.09	0.9-1.4	1.00	0.9-1.3	1.09	0.9-1.3	1.05	0.8-1.3

Reference category: adequate weight gain

uOR, unadjusted odd ratio, univariate analyses; aOR adjusted odds ratio, multivariate analyses, adjusting for maternal age, pre-pregnancy BMI, parity, ethnicity, income, time in Canada, education, number of prenatal visits

## Discussion

This study aimed to ascertain women’s experiences with prenatal counselling on weight gain, nutrition, and diet, particularly in women who did not meet the GWG guidelines. To evaluate the relationship between the adherence to GWG guidelines and the women’s recollection of the counselling received from their health care providers we assessed eight domains of prenatal counselling linked to maintenance of body weight and healthy lifestyle during pregnancy: advice on nutrition, weight gain, working during pregnancy, physical activity, intake of vitamins and minerals, and drugs exposure. The key finding of this study is that, despite most women being comfortable asking questions of their health care provider and recalling satisfactory answers to the inquires, less than two thirds of women recall advice in any of the other domains of lifestyle and behaviour counselling, regardless of their weight gain trajectory. The finding that approximately 40% of women in the study did not recall discussions with the health care provider about GWG in pregnancy suggests that either there was no discussion, or if discussed, it was not memorable enough for women. Furthermore, we report no difference in the prenatal advice received on any of the eight domains of prenatal counselling assessed between women with adequate, inadequate, and excessive GWG. The fact that the recall of prenatal counselling did not differ by GWG suggests that either there may be lack of specific, targeted information based on weight, or the information is not delivered in a memorable fashion. The observation that 33.3% of women entered pregnancy overweight or obese, combined with the finding that 64.1% had non-optimal GWG indicates that broad population health strategies combined with clinical support are required to facilitate improvements in health. Because in our study women with either of these concerns did not differ by income and social strata further supports the need for population level approaches.

Pregnancy-related weight gain is a sensitive subject, due to the common misperception of body weight among women of reproductive age [15, 16], with overweight and obese women more likely underestimating their body weight [14]. Women believe they must gain weight to optimize fetal growth and that the weight will be lost after delivery [26, 27]. The long-standing myth “eating for two” persists and many women are discouraged when they do not easily return to their prepregnant weight [27, 28]. Health care providers should acknowledge these concerns and provide information and support to help women in making positive lifestyle choices and achieve appropriate weight gain [28].

In spite of women feeling comfortable when asking advice, there was no difference in the recollection of the advice women of various GWG received. This suggests that women did not have enough knowledge to pursue the subject with their health care provider or that the counselling was non-specific and/or not personalized to patient’s needs. Emerging evidence shows that provision of adequate and targeted information by prenatal care providers about the optimization of nutrition, lifestyle and weight gain in pregnancy is predictive of meeting GWG guidelines [29]. An increase in the exchange of information, and improvement in general knowledge about the immediate and long-term effects of non-optimal GWG on maternal and fetal health, may facilitate lifestyle and behavioral changes as these conversations become normalized and may be a catalyst for future health throughout later life [11]. One-time counselling and advice for healthy eating and exercise might not help most of the women [30]. Instead, ongoing conversation over the course of pregnancy and adjusted, targeted interventions would be more efficient [31, 32].

There was no difference in the number of encounters with the health care providers, when stratified by GWG. The reasons for our findings may stem from the fact that the practice guidelines do not clearly define the most adequate time or how many times a discussion about the healthy body weight and GWG must take place for the optimum uptake of information. Furthermore, there are no guideline recommendations regarding routine maternal weighing in pregnancy [15]. The guidelines recommend weight management during the periodic health examinations and other appointments for gynaecologic care prior to pregnancy, to ensure women enter pregnancy with a BMI < 30 kg/m2, and ideally, less than 25 kg/m2. We did not assess and consequently cannot exclude that women may have had knowledge about ideal weight to enter pregnancy or recommendations on weight gain in pregnancy from previous health care encounters. Studies have shown that most pregnant women have poor knowledge about obesity and GWG, consequences on pregnancy health and management strategies [33]. Lack of knowledge may limit addressability of these issues successfully during antenatal checks.

We acknowledge that our findings reflect women’s recollection of the encounter with the health care provider and may underestimate the information provided from the clinician. Effective communication between patient and provider is crucial to motivate action and behaviour change [30]. Brief motivational interviewing has been demonstrated as an effective strategy to support patients, and evidence indicates women who perceive the clinician as supportive are more likely to adhere to recommendations [20, 34, 35].

Recommendations for appropriate GWG and lifestyle modifications during pregnancy have changed throughout history as more has become known about this crucial time [12, 17, 36]. Pregnancy obesity guidelines and policies have been developed in many countries where obesity and excessive GWG are common, although official guidelines for maternal weight management in other countries around the world may also be needed [12, 37]. To date, there are no global maternal weight gain guidelines [12, 38]. While the IOM guidelines can be confidently used and are broadly appropriate for various populations, there is a paucity of country specific target GWG based on pre-pregnancy BMI. Fifty-three countries have reported so far having either a formal or informal policy regarding maternal weight [39]. Only 8% of the countries included policies on all 4 fundamental domains of obesity in pregnancy: 1) assessment of maternal weight at the first prenatal visit (90%); 2) monitoring GWG during pregnancy (81%); 3) providing a target GWG and recommendations to women about healthy GWG (62%); 4) returning to a healthy postpartum weight (13%) [39].

The Canadian guidelines on Obesity in Pregnancy include recommendations on counselling of obese pregnant women with BMI > 30 kg/m^2^ on pregnancy risks associated with obesity, weight gain, nutrition, and food choices during prenatal counselling sessions [40]. The guidelines suggest that, ideally, these should be offered prior to pregnancy so that health status of mothers be optimized before conception [40, 41]. The guidelines also stipulate that all women should be encouraged to participate in regular physical exercise during their pregnancy, either regular aerobic and strength-conditioning activities consistent with the joint recommendations by the Society of Obstetricians and Gynaecologists of Canada and the Canadian Society for Exercise Physiology [42]. Whereas our study did explore prenatal advice on diet, GWG, and physical activity, the questions proposed to the participants allowed recollection of any type of advice on the counselling domains and did not specify the type of physical activity or dietary intake. We did not ask women if they received counselling on the safety and pregnancy-related health benefits of physical activity or lifestyle modifications. No objective measures of physical activity during pregnancy were included in our study, which relied on self-reported assessments of physical exercise prior and during pregnancy. Nevertheless, although the obesity in pregnancy guidelines stipulate that pregnant women should to be encouraged to a healthy lifestyle during pregnancy, does not provide specific recommendations on the reasonable goals to maintain a good fitness level throughout pregnancy [42]. Furthermore, the guidelines does not include recommendations stratified by BMI or GWG category, as the role of behavioral therapy and caloric restriction in obese women to prevent excess GWG has not been established [40]. The Exercise in Pregnancy Guideline while recommending regular physical activity during pregnancy to improve or maintain physical fitness and weight management, does not advise on the optimal intensity, frequency and type of exercise, or most effective counselling methods. Further research is needed to study the effects of exercise on obesity in pregnancy and outcomes [43].

There are no studies on the effectiveness of physician counselling related to preventing excessive GWG and only a handful of studies assessed the level of implementation of guidelines into clinical practice [44]. Beside providers’ personal factors, such as difficulties in discussing a sensitive topic and insufficient training, managing a life-long chronic problem in an acute care model, time limitations, few available resources (as the rhythm of increase in obesity prevalence in women of reproductive age worldwide overcomes the resource development), and lack of a team work model for delivery of health care represent barriers in implementing the guidelines [30, 45, 46]. The GWG counselling practices are also dependent on the priority level the health care provider places on the topic, which appears to be influenced by the length of time for appointments, financial compensation, and model of care, midwifery versus medical [44, 47]. The physician alone should not be the single resource to help and advise these women; dietitians, nurses, midwives, behaviorists and exercise therapists may often be in a much better position to provide appropriate advice and counselling [47]. Therefore, more research is needed to identify the provider barrier to addressing GWG not only during pregnancy but also before and after pregnancy, as well as how to overcome those barriers [48].

## Conclusions

Our study suggests there are missed opportunities in knowledge exchange between women and health care providers in the prenatal period targeting prenatal advice to address the needs of women of all weights but specifically those with increased body weight and non-optimal GWG.

Our report calls for improvements in surveillance of GWG and recommends that health professionals adopt and endorse the professional societies guidelines. The report also calls for the guidelines to be largely publicized to increase public awareness to optimize health prior to and during pregnancy and to normalize health behaviours such as exercise and nutrition. In order to help women to achieve the adequate GWG we call for additional prenatal counselling during which health care providers need to offer counselling, strategies, and support for healthy weight gain, physical activity, diet, and nutrition tailored to women’s particular life circumstances.

Addressing the issue of weight gain during pregnancy and its consequences at prenatal counselling may provide benefits well beyond a woman’s immediate well-being, supporting her future pregnancies and a better health status later in life for her and her children.

## Additional files

Additional file 1: **Figure S1.** Distribution of GWG stratified by pre-pregnancy BMI. (DOCX 126 kb)

Additional file 2: **Table S1.** Recall of prenatal counselling in women stratified by maternal demographic and obstetrical characteristics^1^. (DOCX 20 kb)

## Abbreviations

AOF: All Our Families Study
aOR: Adjusted odds ratio
BMI: Body mass index
GWG: Gestational weight gain
IOM: Institute of Medicine
our: Unadjusted odds ratio
SOGC: Society of Obstetricians and Gynecologists of Canada
