# Supplementary material for: Women’s recall of health care provider counselling on gestational weight gain (GWG): a prospective, population-based study
Source: BMC Pregnancy Childbirth. 2019 Apr 25;19:136. doi: 10.1186/s12884-019-2283-x (PMC6485057; doi:10.1186/s12884-019-2283-x)
Supplement: Supplementary file 1 — Figure S1. Distribution of GWG stratified by pre-pregnancy BMI. (DOCX 126 kb) [file 12884_2019_2283_MOESM1_ESM.docx]

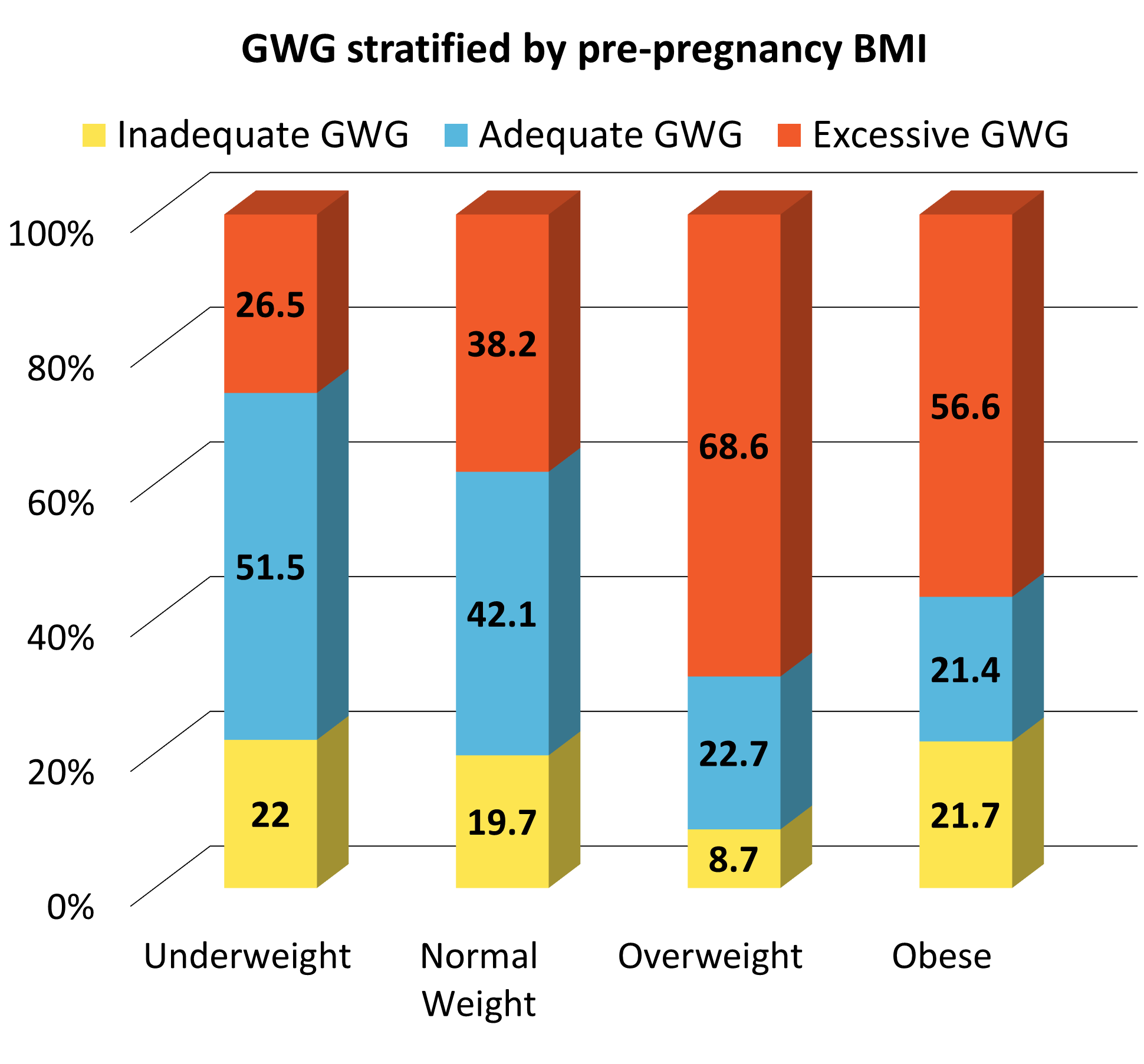


**Additional file 1: Figure S1. Distribution of GWG stratified by pre-pregnancy BMI**

(the numbers on columns represent percentages; percentages add to 100 per column)
