# Supplementary material for: Women’s recall of health care provider counselling on gestational weight gain (GWG): a prospective, population-based study
Source: BMC Pregnancy Childbirth. 2019 Apr 25;19:136. doi: 10.1186/s12884-019-2283-x (PMC6485057; doi:10.1186/s12884-019-2283-x)
Supplement: Supplementary file 2 — Table S1. Recall of prenatal counselling in women stratified by maternal demographic and obstetrical characteristics1. (DOCX 20 kb) [file 12884_2019_2283_MOESM2_ESM.docx]

**Table S1. Recall of prenatal counselling in women stratified by maternal demographic and obstetrical characteristics^1^ n(%)**

| **Maternal demographic and obstetrical characteristics** | **Domains of prenatal counselling** | | | | | | | |
| --- | --- | --- | --- | --- | --- | --- | --- | --- |
|  | **Nutrition** | **Alcohol**  **Consumption** | **Weight gain in pregnancy** | **Vitamins/**  **minerals**  **supplements** | **Exercise**  **and active living** | **Working in pregnancy** | **Non-/prescription**  **drugs** | **Smoking in pregnancy** |
| Pre-pregnancy  BMI  Underweight  Normal weight  Overweight  Obese  **P-value** | 91(68.9)  1260(62.4)  440(68.0)  227(70.5)  **0.836** | 66(50.0)  881(48.7)  321(49.6)  149(46.3)  **0.786** | 84(4.4)  1200(66.4)  425(65.7)  221(68.6)  **0.726** | 110(83.3)  1560(86.3)  551(85.2)  274(85.1)  **0.718** | 73(55.3)  1135(62.8)  415(64.1)  211(65.5)  **0.200** | 56(42.4)  865(47.8)  336(51.9)  173(53.7)  ****0.040*** | 83(62.9)  1082(59.8)  402(22.6)  210(65.2)  **0.266** | 65(49.2)  738(40.8)  281(43.4)  122(37.9)  **0.097** |
| Maternal age  < 35 Years  ≥ 35 Years  **P-value** | 1600(70.3)  369(65.2)  ****0.019*** | 1131(49.7)  250(44.2)  ****0.019*** | 1529(67.1)  360(63.6)  **0.110** | 1958(86.0)  478(84.5)  **0.350** | 1470(64.6)  319(56.4)  ******<0.001*** | 1148(50.4)  248(43.8)  ****0.005*** | 1421(62.4)  317(56.0)  ****0.005*** | 976(42.9)  205(36.2)  **0.097** |
| Education  High school or less  Some/completed  postsecondary  **P-value** | 199(69.8)  1817(69.4)  **0.876** | 144(50.5)  1273(48.6)  **0.538** | 194(68.1)  1734(66.2)  **0.527** | 247(86.7)  2244(85.7)  **0.651** | 175(61.4)  1658(63.3)  **0.527** | 142(49.8)  1287(49.1)  **0.826** | 174(61.1)  1601(61.1)  **0.980** | 156(54.7)  1050(40.1)  ****<0.001*** |
| Household Income  < $60 000  ≥ $60 000  **P-value** | 311(66.6)  1649(70.2)  **0.122** | 196(42.0)  1182(50.3)  ****0.001*** | 283(60.6)  1592(67.8)  ****0.003*** | 390(83.5)  2025(86.2)  **0.128** | 259(55.5)  1522(64.8)  ****<0.001*** | 180(38.5)  1209(51.5)  ****<0.001*** | 253(54.2)  1472(62.7)  ****0.001*** | 204(43.7)  968(42.1)  **0.322** |
| Ethnicity  White/Caucasian  Other  **P-value** | 1619(70.2)  396(66.6)  **0.087** | 1180(51.1)  235(39.5)  ****<0.001*** | 1571(68.1)  356(59.8)  ****<0.001*** | 2002(86.8)  487(81.8)  ****0.002*** | 1510(65.5)  322(54.1)  ****<0.001*** | 1196(51.8)  232(39.0)  ****<0.001*** | 1450(62.9)  323(54.3)  ****<0.001*** | 992(43.0)  212(35.6)  ****0.001*** |
| Time in Canada  Born/lived≥5 years  Lived <5 years  **P-value** | 1832(69.8)  173(64.8)  ****0.001*** | 1296(49.4)  111(41.6)  ****0.015*** | 1773(67.5)  148(55.4)  ****<0.001*** | 2256(85.9)  224(83.9)  **0.370** | 1683(64.1)  144(53.9)  ****0.001*** | 1324(50.4)  98(36.7)  ****<0.001*** | 1617(61.6)  151(56.6)  **0.109** | 1106(42.1)  93(34.8)  ****0.021*** |
| Parity  Nulliparous  Multiparous  **P-value**  Number of prenatal visits  ≤6 prenatal visits  >7 prenatal visits  **P-value** | 1048(74.9)  959(64.2)  ****<0.001***  448(66.7)  1569(72.1)  ****0.007*** | 780(55.7)  628(42.1)  ****<0.001***  318(47.3)  1098(50.4)  **0.158** | 1046(74.7)  873(58.5)  ****<0.001***  442(65.8)  1486(68.3)  **0.229** | 1232(88.0)  1250(83.7)  ****0.001***  567(84.4)  1928(88.6)  ****0.004*** | 998(71.3)  826(55.3)  ****<0.001***  385(57.3)  1448(66.5)  ****<0.001*** | 786(56.1)  634(42.5)  ****<0.001***  282(23.6)  1146(52.6)  ****<0.001*** | 948(67.7)  815(54.6)  ****<0.001***  373(55.5)  1403(64.4)  ****<0.001*** | 675(48.2)  522(35.0)  ****<0.001***  281(41.8)  924(42.4)  **0.773** |

n (%) represents absolute value and percentage of women who answered yes to the survey questions that recalled the prenatal counselling domains

****p-value <0.05*** significant
